# iPathCons and iPathDB: an improved insect pathway construction tool and the database

**DOI:** 10.1093/database/bau105

**Published:** 2014-11-10

**Authors:** Zan Zhang, Chuanlin Yin, Ying Liu, Wencai Jie, Wenjie Lei, Fei Li

**Affiliations:** Department of Entomology, College of Plant Protection, Nanjing Agricultural University and The Key laboratory of Monitoring and Management of Plant Diseases and Insects, Ministry of Agriculture, No. 1, Weigang Road, Xuanwu District, Nanjing, Jiangsu 210095, China

## Abstract

Insects are one of the most successful animal groups on earth. Some insects, such as the silkworm and honeybee, are beneficial to humans, whereas others are notorious pests of crops. At present, the genomes of 38 insects have been sequenced and made publically available. In addition, the transcriptomes of dozens of insects have been sequenced. As gene data rapidly accumulate, constructing the pathway of molecular interactions becomes increasingly important for entomological research. Here, we developed an improved tool, iPathCons, for knowledge-based construction of pathways from the transcriptomes or the official gene sets of genomes. Considering the high evolution diversity in insects, iPathCons uses a voting system for Kyoto Encyclopedia of Genes and Genomes Orthology assignment. Both stand-alone software and a web server of iPathCons are provided. Using iPathCons, we constructed the pathways of molecular interactions of 52 insects, including 37 genome-sequenced and 15 transcriptome-sequenced ones. These pathways are available in the iPathDB, which provides searches, web server, data downloads, etc. This database will be highly useful for the insect research community.

**Database URL:** http://ento.njau.edu.cn/ipath/

## Introduction

Insects are one of the most successful animal groups on earth. They comprise more than a million species, representing about half of all known living organisms. Some insects, such as silkworm (*Bombyx mori*) and honeybee (*Apis mellifera*) are beneficial to humans by producing valuable products and/or services (silk, honey, pollination). In contrast, other species damage crops by feeding on leaves or fruits, causing huge economic losses.

As the sequencing cost has dramatically declined in recent decade, gene sequences data have accumulated rapidly in insects. The genome sequences of 38 insect species have been reported, including 12 species of *Drosophila* (1, 2), seven kinds of ants (3–8), three wasps (9), three mosquitoes (10–12), two butterflies (13, 14), the human body louse *Pediculus humanus humanus* (15), the kissing bug *Rhodnius prolixus* (16), the tsetse fly *Glossina morsitans* (16), a tick *Ixodes scapularis* (16), the honeybee *A mellifera* (17), the silkworm *B. mori* (18), stick insect *Timema cristinae* (19) and several agricultural insect pests, such as the red flour beetle *Tribolium castaneum* (20), pea aphid *Acyrthosiphon pisum* (21), diamondback moth *Plutella xylostella* (22) and locust *Locusta migratoria* (23). In addition, dozens of insects have been sequenced for their transcriptome (the SRA database, September 2014).

Constructing the pathway of molecular interaction from the insect genomes or transcriptomes is important for gene function analysis. Large-scale gene expression analysis is an efficient and widely used technique in molecular biology experiment. However, selecting the right candidate genes for experiment validation is still a challenge. One solution is to find differently expressed genes in a related pathway. To construct pathways, several knowledge-based methods were developed, such as PANTHER (24), Gene Ontology (25), Kyoto Encyclopedia of Genes and Genomes (KEGG) Orthology (KO) (26), Reactome (27) and PharmGKB (28). Although these methods can be applied to the insect gene data, insect pathway construction is still a difficult work. First, most insects have a heterozygous genome, reducing the quality of genome assembly and annotation and increasing the difficulty in pathway construction. Second, compared with mammals and other groups of animals, insects are species-rich and have high evolution diversity. Here, we developed an improved tool for insect pathway construction and built an insect pathway database, which should be helpful to the entomological community.

## Data resources

### Official gene sets of 37 insect species

The genome sequences of 37 insects were downloaded from the NCBI (National Center for Biotechnology Information) genome database or in species-specific databases, such as FlyBase (29), AphidBase (30), BeeBase, ButterflyBase (31), BeetleBase (32), DBM-DB (33), MonarchBase (34), NasoniaBase, HessianFlyBase, ManducaBase, Ant genome (35), VectorBase (36), SilkDB (37) and ChiloDB. When we prepared this article, the genome of locust was published (23). We did not include locust in this work. We downloaded the official gene sets (OGS) of 37 insects (Supplementary Table S1), including *Aedes aegypti* (v2.2), *Anopheles gambiae* (v3.8), *Anopheles darlingi* (v2.2), *Anopheles stephensi* (v2.2), *Culex quinquefasciatus* (v1.4), *G. morsitans* (v1.3), *Lutzomyia longipalpis* (v1.1), *Pediculus humanus corporis* (v1.3), *Phlebotomus papatasi* (v1.1), *Rh. prolixus* (v1.1), *Acromyrmex echinatior* (v3.8), *Atta cephalotes* (v1.2), *Camponotus floridanus* (v3.3), *Harpegnathos saltator* (v3.3), *Linepithema humile* (v1.2), *Pogonomyrmex barbatus* (v1.2), *Solenopsis invicta* (v2.2.3), *A. mellifera* (v3.2), *Nasonia vitripennis* (v1.2), *Drosophila ananassae* (v1.3), *Drosophila erecta* (v1.3), *Drosophila grimshawi* (v1.3), *Drosophila melanogaster* (v5.54), *Drosophila mojavensis* (v1.3), *Drosophila persimilis* (v1.3), *Drosophila pseudoobscura* (v3.1), *Drosophila sechellia* (v1.3), *Drosophila simulans* (v1.4), *Drosophila virilis* (v1.2), *Drosophila willistoni* (v1.3), *Drosophila yakuba* (v1.3), *Danaus plexippus* (v2.0), *Heliconius melpomene* (v1.1), *B. mori* (v2.0), *T. castaneum* (v3.0), *Ac. pisum* (v2.1b) and *Ti. cristinae* (v1.0).

### Insect transcriptomes of 15 insects

We downloaded the RNA-seq raw data of 15 insects from Sequence Read Archive database (SRA, http://www.ncbi.nlm.nih.gov/sra/). The genomic data of these species are not available at present. They are *A. cerana*, *Lucilia sericata*, *Rhagoletis pomonella*, *Aedes albopictus*, *Galleria mellonella*, *Chilo suppressalis*, *Spodoptera exigua*, *Manduca sexta*, *Melitaea cinxia*, *P. xylostella*, *Zygaena filipendulae*, *Dendroctonus ponderosae*, *Nilaparvata lugens*, *Oncopeltus fasciatus* and *Bemisia tabaci.* The SRA accession numbers are given in Supplementary Table S2. The transcriptome of *C. quinquefasciatus* was also downloaded and used to estimate the precision and coverage of the iPathCons.

The SRA database only provides the sequences of raw reads. For most insects, the assembled transcriptomes are not available. Therefore, we assembled these 15 transcriptomes *de novo*. The statistic of assembled transcriptome was presented in the Supplementary Table S3. First, the raw data were cleaned by removing adaptor sequences, empty reads, and low-quality reads that contain *N* or whose average nucleotides quality is less than 15. Second, we merged the raw reads from different samples of same species to obtain contigs as many as possible. Third, Trinity was used to assemble Illumina Solexa raw reads with default parameters (38). The insects included *A. cerana*, *L. sericata*, *Ch. suppressalis*, *P. xylostella*, *N. lugens*, *Be. tabaci* and *C. quinquefasciatus*. The Newbler was used to assemble Roche/454 raw data with default parameters, including *Sp. exigua*, *R. pomonella*, *Ae. albopictus, De. ponderosae, O. fasciatus* and *M. sexta*. For the EST (Expressed Sequence Tag) data of *Ga. mellonella*, *Me. cinxia,* and *Z. filipendulae*, we assembled the transcripts using the Cap3 software (39). Finally, the assembled transcripts were annotated using BLASTX (Basic Local Alignment Search Tool) against the NCBI nr database.

### KEGG data

The KEGG database provides the most commonly used resources for pathway analysis (40). KEGG contains the genes of 21 insects (Table 1), which were downloaded from the NCBI RefSeq database (41). Among these 21 insects, 14 were from Diptera, including 11 *Drosophila* species and three mosquitoes. The KEGG Markup Language (KGML) is a format of the KEGG pathway maps, which can be used to draw KEGG pathways and to model gene and chemical networks. Given that KEGG requires a subscription to its FTP server, we downloaded the KGML files from their individual web download pages.

**Table 1.** Data statistic of iPathDB

Order	Species	Nucleotide or protein sequences	Annotated sequences	KO term	Pathway
Coleoptera	*De. ponderosae*^¤^	25 360	7166	1997	261
	T. castaneum*	16 733	2093	2093	256
Diptera	Aedes aegypti*	15 786	2097	2097	257
	*Ae. albopictus*^¤^	48 422	15 794	2013	253
	*Anopheles darlingi*^⊕^	11 430	4309	1849	187
	Anopheles gambiae*	12 811	2130	2130	258
	*Anopheles stephensi*^⊕^	23 287	4980	1867	189
	C. quinquefasciatus*	18 955	2068	2068	259
	D. ananassae*	15 143	2087	2087	257
	D. erecta*	15 123	2089	2088	256
	D. grimshawi*	15 066	2095	2094	257
	D. melanogaster*	29 453	2159	2156	258
	D. mojavensis*	14 665	2082	2082	257
	D. persimilis*	16 948	2037	2036	254
	D. pseudoobscura*	16 929	2072	2072	256
	D. sechellia*	16 544	2073	2073	251
	D. simulans*	15 486	1984	1983	254
	D. virilis*	14 567	2090	2089	257
	D. willistoni*	15 596	2079	2079	257
	D. yakuba*	16 158	2102	2101	257
	*G. morsitans*^⊕^	12 457	4726	1658	182
	*L. sericata*^¤^	146 250	45 768	2272	263
	*Lu. longipalpis*^⊕^	10 110	3946	1483	176
	*Ph. papatasi*^⊕^	11 164	4388	1506	178
	*R. pomonella*^¤^	13 071	3157	1032	223
Hemiptera	A. pisum*	36 198	2046	2046	252
	*Be. tabaci*^¤^	54 860	7161	2078	255
	*N. lugens*^¤^	36 748	9475	2163	258
	*O. fasciatus*^¤^	4863	922	520	171
	*Rh. prolixus*^⊕^	15 441	4579	1580	178
Hymenoptera	*A. cerana cerana*^¤^	50 373	11 143	2150	260
	A. mellifera*	15 319	1981	1981	253
	*At. cephalotes*^⊕^	18 093	5424	1957	187
	*Acromyrmex echinatior*^⊕^	17 278	5651	1957	187
	*C. floridanus*^⊕^	17 064	5365	1959	187
	*H. saltator*^⊕^	18 564	5880	1964	187
	*Li. humile*^⊕^	16 116	5257	1966	187
	Na. vitripennis*	18 900	1968	1968	256
	*Po. barbatus*^⊕^	17 189	5248	1963	187
	*So. invicta*^⊕^	16 522	5104	1793	187
Lepidoptera	B. mori*	14 623	2138	2467	296
	*Ch. suppressalis*^¤^	37 040	7005	2029	256
	*D. plexippus*^⊕^	15 130	5111	1648	184
	*Ga. mellonella*^¤^	40 035	16 314	1675	255
	*He. melpomene*^⊕^	12 829	4493	1580	181
	*M. sexta*^¤^	67 902	1213	744	189
	*Me. cinxia*^¤^	195 086	7621	1598	249
	*P. xylostella*^¤^	456 026	93 941	2330	266
	*Sp. exigua*^¤^	153 619	31 071	1957	260
	*Z. filipendulae*^¤^	42 735	11 709	1861	254
Phthiraptera	Pe. humanus corporis*	10 773	2087	2087	256
Phasmatodea	*Ti. cristinae*^⊕^	17 0931	56 536	2073	260

*21 genome-sequenced and KEGG-annotated insects; ^⊕^16 genome-sequenced insects; ^¤^15 transcriptome-sequenced insects.

## iPathCons

**Figure 1.**
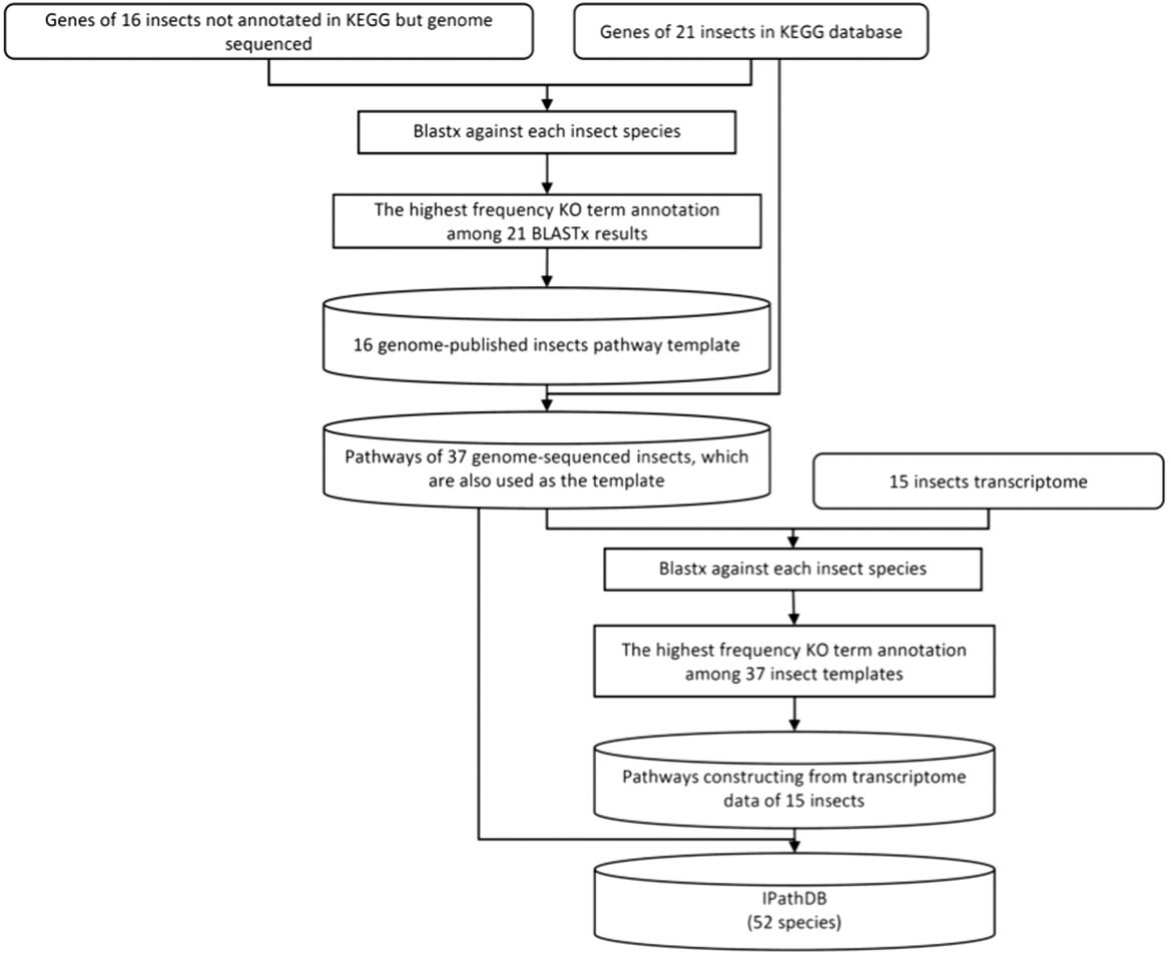
The pipeline of iPathCons.

We developed a pipeline, iPathCons, to construct insect pathways using either OGSs or transcriptomes (Figure 1).

### Data preparation

We downloaded the KEGG genes of 21 genome-sequenced insects, the OGS of 37 genome-sequenced insects and the transcriptome data of 15 species. For the 21 KEGG-annotated insects with OGS data, we compared the sequences in KEGG gene data and OGS data sets, (i) if there is a length difference, we used the long transcript; (ii) we kept those genes even they appear in only one gene data set.

### KO assignment

Assigning KO terms is a crucial step in pathway construction. We used a voting system for KO assignment in the iPathCons. The protein data sets of 21 KEGG-annotated insects were divided by species. The protein sequences of each insect were formatted to build the local BLAST database, respectively, which were used as the template for KO assignment of other 16 genome-sequenced insects. For each insect need be annotated, its protein sequences were used to BLASTP against the template of every KEGG-annotated insect. The best BLASTP hit was used to assign KO terms (*E*-value ≤ 10^−5^), which has been widely used in the KOBAS (42), KAAS (43) and Blast2GO (44, 45). In this way, every protein was assigned KO terms for 21 times. The term that appears at the highest frequency (the minimum cutoff is ≥ 2) was used as the final KO assignment for the protein sequences.

A similar procedure was used to deduce pathway from the transcriptomes of 15 insects. All 37 genome-sequenced insects, including 21 KEGG-annotated and 16 iPathCons-annotated ones, were used as the template for KO assignment. The protein sequences of each genome-sequenced insect were used as the local BLAST database, respectively. The transcriptome sequences were used to BLASTP against the local BLAST database (*E*-value ≤ 10^−5^). The KO term that appeared at the highest frequency (the minimum cutoff is ≥ 2) was used as the final KO annotation.

## Validation of iPathCons

We used *C. quinquefasciatus* gene data to validate the iPathCons. The protein sequences of *C. quinquefasciatus* have been annotated by the KEGG database. We removed all protein sequences of *C. quinquefasciatus* from the KEGG template and used them to deduce pathways using the iPathCons. The results indicated that the precision reached 95% and the coverage was 94% (*E*-value ≤ 10^−5^). We also used the transcriptome data of *C. quinquefasciatus* for pathway construction and obtained a similar result.

We compared the results of the iPathCons with that of other relate tool KAAS, which is a widely used pathway annotation tool provided by the KEGG database. The transcriptome of *A. cerana cerana* and *Ga. mellonella* were used to deduce pathways by both iPathCons and KAAS. The results indicated that similar number of KO terms and pathways were annotated in *A. cerana cerana* by two tools, whereas the iPathCons found 1675 KO terms and 255 pathways, much more than 1511 KO terms and 239 pathways annotated by the KAAS (Table 2). The iPathCons annotated significantly more contigs than the KAAS, possibly because much more templates were used in the iPathCons. However, it should be noticed that both iPathCons and KAAS relied on homology analysis to deduce the pathway. So, the results should have some false positive and need to be confirmed by molecular experiments.

**Table 2.** Comparison of iPathCons and KAAS

	*A. cerana cerana*		*Ga. mellonella*	
	iPathCons	KAAS	iPathCons	KAAS
Total nucleotide sequences	50 373		40 035	
Annotated sequences	11 143	3469	16 314	2083
KO term	2150	2146	1675	1511
Pathway	260	254	255	239

## Availability of iPathCons software

Both stand-alone software and the web server were provided. The stand-alone, command-line program was written using Perl language. The program consists of three parts: the main program, the ‘doc’ folder containing the index of K number and KO terms, the ‘db’ folder containing the local BLAST database. iPathCons can complete the following tasks: (i) annotating an insect transcriptome or gene sets for pathway construction; (ii) generating KGML files that can be opened by VANTED (22) and KEGG-ED (23); and (iii) generating links for each pathway showing the KEGG pathways.

## Database construction by iPathDB

### Database system implementation

**Figure 2.**
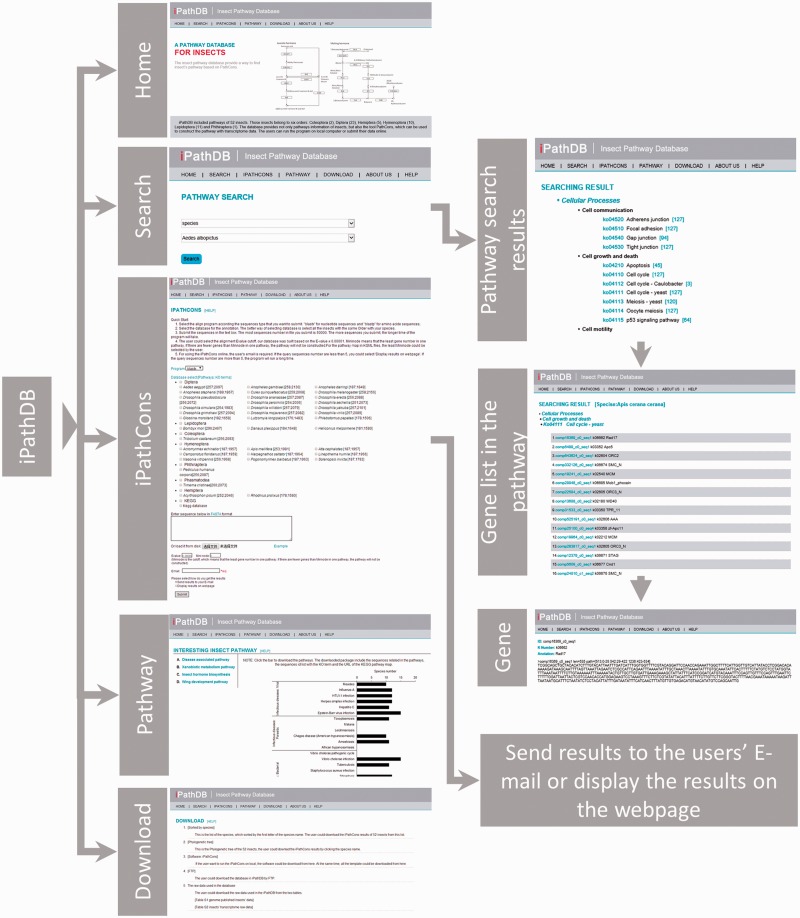
Overview of iPathDB web pages.

We constructed an insect pathway database named as the iPathDB, which was developed on a Linux operating system (Redhat 5.6, Raleigh, NC, USA). The Apache HTTP server was used to handle queries from web clients through PHP scripts to perform searches. The web pages were written using html, PHP, CSS and JavaScript. The architecture of iPathDB is presented in Figure 2.

### Search

Users can search insect pathways using keywords for species, pathway ID and pathway name. When using species name as the search keyword, all pathways for that species will be presented. When using pathway ID or pathway name as the search keyword, the pathway will be given for all species in the database. Search results provide gene sequences, annotations and a pathway map.

### Online server

An online iPathCons server was provided. The KEGG- and iPathCons-annotated gene sets from different insect orders, including Diptera, Lepidoptera, Coleoptera, Hymenoptera, Phthiraptera and Hemiptera, are used as the template for constructing insect pathways. Users can select a template according to their requirements. When the queried sequences are less than 10, the results are displayed in the Webpage directly. If the queried sequences are more than 10, a URL link of the iPathCons results will be sent to the user via e-mail.

### Download page

Both FTP and HTTP download options are provided. The iPathDB FTP site is ftp://ftp.insect-genome.com/pub/iPathDB/. On the download page, insect pathway information classified by species and the stand-alone version of iPathCons can be downloaded. A phylogenetic tree is provided to show evolutionary relationships. The insect pathway files can be directly downloaded by clicking the links in the tree. On the FTP server, insect pathway information is provided by species. The zipped archive contains a readme file, sequences in FASTA format, KEGG map links, a KGML file, a pathway summary and a pathway list. In total, iPathDB contains 11 581 pathways from 52 species and 387 478 annotated sequences (Table 1).

## Insect pathways

### Disease-associated pathways

**Figure 3.**
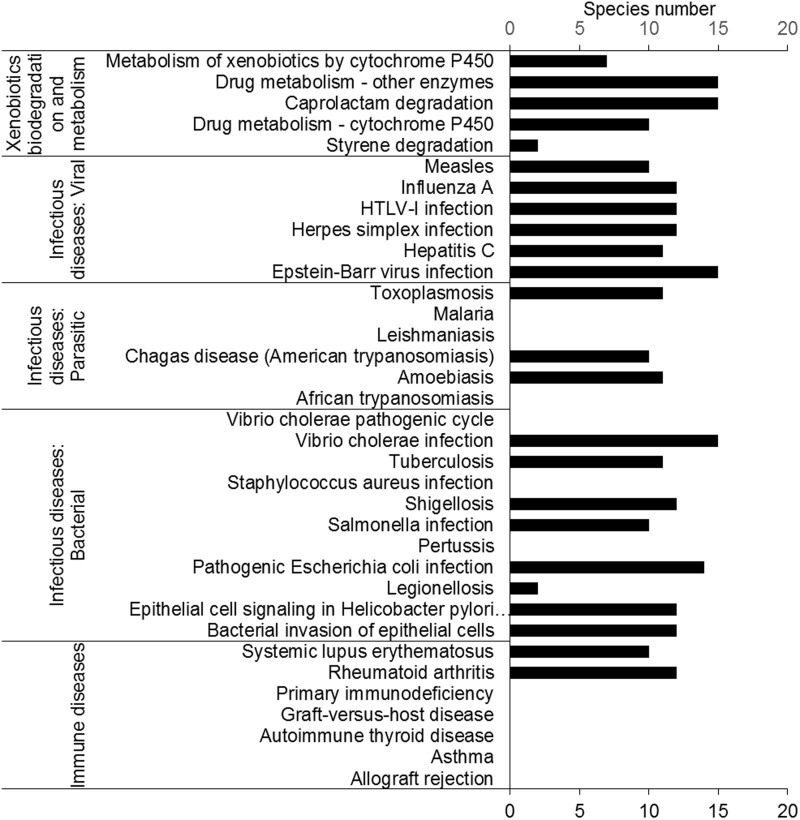
Human-disease and insecticide-resistance pathways. Most pathways in the ‘immune disease’ category were not found. Five pathways involved in insecticide resistance were found in the insect transcriptomes.

Insects have been studied to model human diseases. Insect disease models can provide an efficient way to study mechanisms and screen drugs. Interestingly, the results showed that 72% of human disease pathways could be found in insects (Figure 3). In total, 17 human disease-associated pathways were found in insects, including bacterial, viral and parasitic infectious disease. In contrast, only two ‘immune disease’ pathways were found, suggesting that the immune systems of insects and humans are quite different. These results suggested that insects are good candidates for modeling human infectious diseases. A successful example is that of *D. melanogaster*, which has been used to model cholera (46).

### Xenobiotic metabolism pathways

Most insects feed on plants. To protect themselves, plants produce many kinds of secondary metabolites. Insect herbivores have evolved many of xenobiotic degradation and metabolism pathways in response. Almost all insects have the pathways belonging to the category ‘xenobiotics biodegradation and metabolism’. We found that all insects contained the ‘caprolactam degradation’ pathway. Caprolactam is a pesticide intermediate (Figure 3).

### Signaling pathways

Signaling pathways are important signal transduction pathways related with proteins that pass signals from outside of a cell to the inside of the cell. In total, 29 signal pathways were found. The well-studied important signal pathways exist in almost all 52 insects, including Toll-like receptor signal pathway, MAPK signal pathway, NFKB signal pathway, Notch signal pathway, etc. This suggests that these pathways are highly conserved and also play important functions in insects.

### Insect hormone biosynthesis

**Figure 4.**
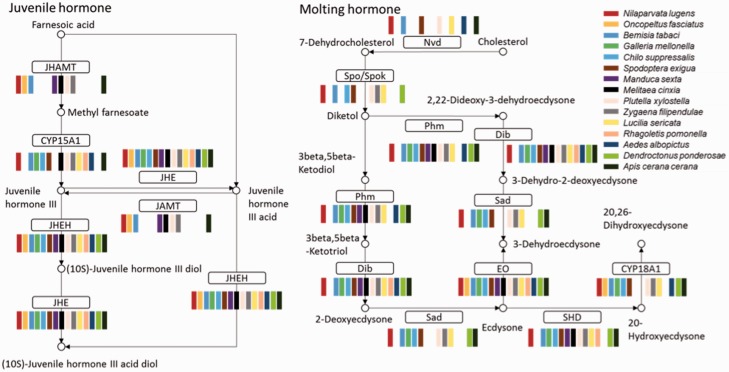
Insect hormone biosynthesis pathway. Most of the enzymes in the pathway could be found in all 15 insect transcriptomes; JHEH, JHE, and EO were found in all 15. Abbreviations: JHAMT, juvenile hormone acid methyltransferase; JHE, juvenile-hormone esterase; JHEH, juvenile hormone epoxide hydrolase; Nvd, cholesterol 7-dehydrogenase; Spo/Spok, CYP307A; Phm, CYP306A1; ecdysteroid 25-hydroxylase; Dib, CYP302A1, ecdysteroid 22-hydroxylase; Sad, ecdysteroid 2-hydroxylase; EO, ecdysone oxidase; SHD, ecdysone 20-monooxygenase.

Almost all insects undergo incomplete metamorphosis from immature nymphs, which resemble the adults, or complete metamorphosis from immature larvae, which are significantly different from the adult. Both molting and juvenile hormone control the insect metamorphosis. Hormone biosynthesis pathways were identified in all 52 insects. In the genome-sequenced insects, almost all genes in the insect hormone biosynthesis pathway were found, suggesting that this pathway is highly conserved in insects. All insects with transcriptome data had juvenile hormone epoxide hydrolase, juvenile-hormone esterase and ecdysone oxidase. Ecdysteroid 25-hydroxylase, CYP306A1 (Phm), ecdysteroid 22-hydroxylase and CYP302A1 (Dib) were found in almost all insects (Figure 4). We compared the pathway members between holometabous and hemimetabolous insects, finding no apparent difference from present data. A detail analysis of the pathway differences is worthy of further investigation. Because of the low quality of the insect transcriptome data, some genes in the insect hormone biosynthesis pathway were missing. The completeness of this pathway can be used as a parameter to estimate the quality of genome annotation or transcriptome assembly.

### Wing development pathway

**Figure 5.**
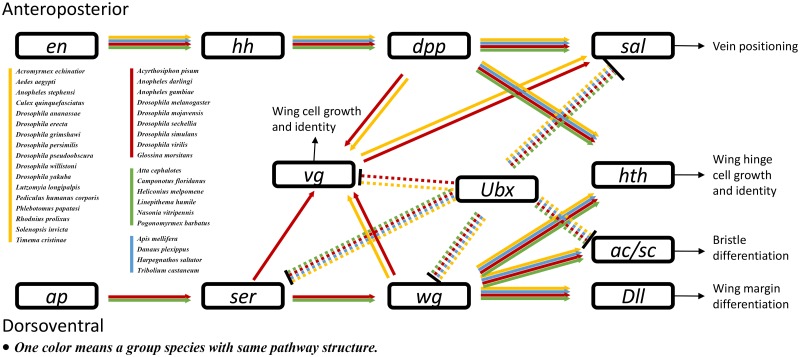
Insect wing development pathway. Arrowheads and bars indicate activation and repression, respectively. Dashed lines indicate Ubx-related regulation specific to fly halteres. Abbreviations: *en*, *engrailed*; *hh*, *hedgehog*; *dpp*, *decapentaplegic*; *sal*, *spalt major*; *Ubx*, *Ultrabithorax*; *vg*, *vestigial*; *hth, homothorax*; *ap, apterous; Ser, Serrate*; *wg*, *wingless*; *Dll*, *Distalless*; *ac/sc*, *achaete/scute*.

Insects are characterized by having six legs and four wings, which enable diverse mobile abilities. Insect wing development is an important research topic. However, no wing development pathway is available in the KEGG or other gene network databases. Therefore, we constructed a wing development pathway after reference mining research on wing development in *D. melanogaster* (47–57) and *Ac. pisum* (58). KGML files of wing development pathways in these two species were produced. Then, those files were used as templates to construct the pathways in other species (Figure 5). To best of our knowledge, this is the first report of an insect wing development pathway. The results indicated that almost all insects have genes in this pathway. However, major parts of genes associated with wing development were missing in the flightless silkworm, *B. mori*. Since the silkworm has been domesticated for thousands of years, the impact of domestication on the evolution of wing development requires further investigation.

## Conclusion

We developed an improved analysis tool for constructing insect pathways. Both stand-alone software and web servers are provided. Users can construct insect pathways from a list of genes. An insect pathway database was also built that contains well-annotated insect pathways from 52 species.

## Future study

1. Knowledge-based construction of insect pathways relies on sequence data. Therefore, we will continually update iPathDB by adding more insect genomes once they are sequenced and published. We will also reconstruct the pathway when new versions of OGSs are released.
2. Evolutionary analysis of insect pathway is an interesting topic that is worthy of further investigation. In the future, as more reliable insect pathways are added to iPathDB, we will carry out insect pathway conservation analysis. IPathDB will display conserved insect pathways in various insect species.

## Supplementary Data

Supplementary data are available at *Database* Online.

## Funding

This work was supported by the National High Technology Research and Development Program (‘863’Program) of China (2012AA101505), the National Science Foundation of China (31171843, 31301691) and the Jiangsu Science Foundation for Distinguished Young Scholars (BK2012028). Funding for open access charge: BK2012028.

*Conflict of interest*. None declared.

## Supplementary Material

Supplementary Data
