# Supplementary material for: iPathCons and iPathDB: an improved insect pathway construction tool and the database
Source: Database (Oxford). 2014 Nov 10;2014:bau105. doi: 10.1093/database/bau105 (PMC4227299; doi:10.1093/database/bau105)
Supplement: Supplementary Data [file supp_2014_bau105_index.html]

iPathCons and iPathDB: an improved insect pathway construction tool and the database — Supplementary Data 

# iPathCons and iPathDB: an improved insect pathway construction tool and the database

## Supplementary Data

files

**Files in this Data Supplement:**

- Supplementary Data - doc file
- Supplementary Data - doc file
- Supplementary Data - doc file
