# Supplementary material for: iPathCons and iPathDB: an improved insect pathway construction tool and the database
Source: Database (Oxford). 2014 Nov 10;2014:bau105. doi: 10.1093/database/bau105 (PMC4227299; doi:10.1093/database/bau105)
Supplement: Supplementary Data [file supp_bau105_Supplementary_Table_1.doc]

**Table S1 genome published insects’ data**

| Order | Species | URL |
| --- | --- | --- |
| Hymenoptera | *Apis mellifera* | http://hymenopteragenome.org/beebase/sites/hymenopteragenome.org.beebase/files/data/consortium_data/amel_OGSv3.2_pep.fa.gz |
|  | *Nasonia vitripennis* | http://hymenopteragenome.org/nasonia/nasonia_genome_consortium/data/Nvit_OGSv1.2_pep.fa.gz |
|  | *Atta cephalotes* | http://hymenopteragenome.org/drupal/sites/hymenopteragenome.org.atta/files/data/acep_OGSv1.2_pep.fa.gz |
|  | *Pogonomyrmex barbatus* | http://hymenopteragenome.org/drupal/sites/hymenopteragenome.org.pogo/files/data/pbar_OGSv1.2_pep.fa.gz |
|  | *Solenopsis invicta* | http://hymenopteragenome.org/drupal/sites/hymenopteragenome.org.solenopsis/files/data/sinv_OGSv2.2.3_pep.fa.gz |
|  | *Harpegnathos saltator* | http://hymenopteragenome.org/drupal/sites/hymenopteragenome.org.harpegnathos/files/data/hsal_OGSv3.3_pep.fa.gz |
|  | *Camponotus floridanus* | http://hymenopteragenome.org/drupal/sites/hymenopteragenome.org.camponotus/files/data/cflo_OGSv3.3_pep.fa.gz |
|  | *Acromyrmex echinatior* | http://hymenopteragenome.org/drupal/sites/hymenopteragenome.org.acromyrmex/files/data/aech_OGSv3.8_pep.fa.gz |
|  | *Linepithema humile* | http://hymenopteragenome.org/drupal/sites/hymenopteragenome.org.linepithema/files/data/lhum_OGSv1.2_pep.fa.gz |
| Coleoptera | *Tribolium castaneum* | ftp://ftp.bioinformatics.ksu.edu/pub/BeetleBase/3.0/Sequences/Manually_Curated_CDS_Peptide_mRNA/Manually_Curated_Peptide/ |
| Lepidoptera | *Danaus plexippus* |  |
|  | *Bombyx mori* | ftp://silkdb.org/pub/current/Gene/Glean_genes/silkworm_glean_pep.fa.tar.gz |
|  | *Heliconius melpomene* | http://butterflygenome.org/sites/default/files/Hmel1-1_Release_20120601.tgz |
| Phthiraptera | *Pediculus humanus corporis* | https://www.vectorbase.org/download/pediculus-humanus-usdapeptidesphumu21fagz |
| Phasmatodea | *Timema knulli* | http://nosil-lab.group.shef.ac.uk/wp-content/uploads/data/timema_cristinae_draft_2014_gff_prots.tar.bz2 |
| Hemiptera | *Acyrthosiphon pisum* | https://www.aphidbase.com/aphidbase/content/download/3347/34150/file/aphidbase_2.1b_pep.fasta.bz2 |
|  | *Rhodnius prolixus* | https://www.vectorbase.org/download/rhodnius-prolixus-cdcpeptidesrproc12fagz |

**Table S1 genome published insects’ data (continued)**

| Order | Species | URL |
| --- | --- | --- |
| Diptera | *Drosophila melanogaster* | ftp://ftp.flybase.net/genomes/Drosophila_melanogaster/dmel_r5.54_FB2013_06/fasta/dmel-all-translation-r5.54.fasta.gz |
|  | *Drosophila pseudoobscura* | ftp://ftp.flybase.net/genomes/Drosophila_pseudoobscura/dpse_r3.1_FB2013_02/fasta/dpse-all-translation-r3.1.fasta.gz |
|  | *Drosophila ananassae* | ftp://ftp.flybase.net/genomes/Drosophila_ananassae/dana_r1.3_FB2011_08/fasta/dana-all-translation-r1.3.fasta.gz |
|  | *Drosophila erecta* | ftp://ftp.flybase.net/genomes/Drosophila_erecta/dere_r1.3_FB2011_08/fasta/dere-all-translation-r1.3.fasta.gz |
|  | *Drosophila persimilis* | ftp://ftp.flybase.net/genomes/Drosophila_persimilis/dper_r1.3_FB2010_02/fasta/dper-all-translation-r1.3.fasta.gz |
|  | *Drosophila sechellia* | ftp://ftp.flybase.net/genomes/Drosophila_sechellia/dsec_r1.3_FB2010_02/fasta/dsec-all-translation-r1.3.fasta.gz |
|  | *Drosophila simulans* | ftp://ftp.flybase.net/genomes/Drosophila_simulans/dsim_r1.4_FB2012_03/fasta/dsim-all-translation-r1.4.fasta.gz |
|  | *Drosophila willistoni* | ftp://ftp.flybase.net/genomes/Drosophila_willistoni/dwil_r1.3_FB2010_02/fasta/dwil-all-translation-r1.3.fasta.gz |
|  | *Drosophila yakuba* | ftp://ftp.flybase.net/genomes/Drosophila_yakuba/dyak_r1.3_FB2011_08/fasta/dyak-all-translation-r1.3.fasta.gz |
|  | *Drosophila grimshawi* | ftp://ftp.flybase.net/genomes/Drosophila_grimshawi/dgri_r1.3_FB2010_02/fasta/dgri-all-translation-r1.3.fasta.gz |
|  | *Drosophila mojavensis* | ftp://ftp.flybase.net/genomes/Drosophila_mojavensis/dmoj_r1.3_FB2011_05/fasta/dmoj-all-translation-r1.3.fasta.gz |
|  | *Drosophila virilis* | ftp://ftp.flybase.net/genomes/Drosophila_virilis/dvir_r1.2_FB2012_01/fasta/dvir-all-translation-r1.2.fasta.gz |
|  | *Glossina morsitans* | https://www.vectorbase.org/download/glossina-morsitans-yaletranscriptsgmory14fagz |
|  | *Anopheles gambiae* | https://www.vectorbase.org/download/anopheles-gambiae-pestpeptidesagamp42fagz |
|  | *Aedes aegypti* | https://www.vectorbase.org/download/aedes-aegypti-liverpoolpeptidesaaegl32fagz |
|  | *Culex quinquefasciatus* | https://www.vectorbase.org/download/culex-quinquefasciatus-johannesburgpeptidescpipj22fagz |
|  | *Anopheles darlingi* | https://www.vectorbase.org/download/anopheles-darlingi-coaripeptidesadarc32fagz |
|  | *Anopheles stephensi* | https://www.vectorbase.org/download/anopheles-stephensi-indianpeptidesastei22fagz |
|  | *Lutzomyia longipalpis* | https://www.vectorbase.org/download/lutzomyia-longipalpis-jacobinapeptidesllonj11fagz |
|  | *Phlebotomus papatasi* | https://www.vectorbase.org/download/phlebotomus-papatasi-israelpeptidesppapi11fagz |
