# Supplementary material for: iPathCons and iPathDB: an improved insect pathway construction tool and the database
Source: Database (Oxford). 2014 Nov 10;2014:bau105. doi: 10.1093/database/bau105 (PMC4227299; doi:10.1093/database/bau105)
Supplement: Supplementary Data [file supp_bau105_Supplementary_Table_2.doc]

**Table S2 insects’ transcriptome raw data**

| Order | Species | Sample | SRA accession number | URL |
| --- | --- | --- | --- | --- |
| Hymenoptera | *Apis cerana cerana* | Egg, larvae, pupa and adult | SRR536773 | ftp://ftp-trace.ncbi.nlm.nih.gov/sra/sra-instant/reads/ByRun/sra/SRR/SRR536/SRR536773/SRR536773.sra |
| Lepidoptera | *Galleria mellonella* | Larvae | ERP000555 | http://www.ncbi.nlm.nih.gov/sra/?term=ERP000555 |
|  | *Chilo suppressalis* | Midgut | SRR449559 | ftp://ftp-trace.ncbi.nlm.nih.gov/sra/sra-instant/reads/ByRun/sra/SRR/SRR449/SRR449559/SRR449559.sra |
|  | *Spodoptera exigua* | Larvae | SRR413990 | ftp://ftp-trace.ncbi.nlm.nih.gov/sra/sra-instant/reads/ByRun/sra/SRR/SRR413/SRR413990/SRR413990.sra |
|  | *Manduca sexta* | Antennae | ERP000526 | http://www.ncbi.nlm.nih.gov/sra/?term=ERP000526 |
|  | *Melitaea cinxia* | Larvae, pupae and adults | SRA000207 | http://www.ncbi.nlm.nih.gov/sra/?term=SRA000207 |
|  | *Plutella xylostella* | Egg | SRR179062 | ftp://ftp-trace.ncbi.nlm.nih.gov/sra/sra-instant/reads/ByRun/sra/SRR/SRR179/SRR179062/SRR179062.sra |
|  |  | Larvae | SRR179508 | ftp://ftp-trace.ncbi.nlm.nih.gov/sra/sra-instant/reads/ByRun/sra/SRR/SRR179/SRR179508/SRR179508.sra |
|  |  | Pupa | SRR179509 | ftp://ftp-trace.ncbi.nlm.nih.gov/sra/sra-instant/reads/ByRun/sra/SRR/SRR179/ SRR179509/ SRR179509.sra |
|  |  | Adult | SRR179510 | ftp://ftp-trace.ncbi.nlm.nih.gov/sra/sra-instant/reads/ByRun/sra/SRR/SRR179/ SRR179510/ SRR179510.sra |
|  |  | Chlorpyrifos-resistant larvae | SRR179511 | ftp://ftp-trace.ncbi.nlm.nih.gov/sra/sra-instant/reads/ByRun/sra/SRR/SRR179/ SRR179511/ SRR179511.sra |
|  |  | Fipronil-resistant larvae | SRR179587 | ftp://ftp-trace.ncbi.nlm.nih.gov/sra/sra-instant/reads/ByRun/sra/SRR/SRR179/ SRR179587/ SRR179587.sra |
|  | *Zygaena filipendulae* | Larvae removed gut | SRR023844 | ftp://ftp-trace.ncbi.nlm.nih.gov/sra/sra-instant/reads/ByRun/sra/SRR/SRR023/SRR023844/SRR023844.sra |

**Table S2 insects’ transcriptome raw data (continued)**

| Order | Species | Sample | SRA accession number | URL |
| --- | --- | --- | --- | --- |
| Diptera | *Lucilia sericata* | 0-1d pupa | SRR350017 | ftp://ftp-trace.ncbi.nlm.nih.gov/sra/sra-instant/reads/ByRun/sra/SRR/SRR350/SRR350017/SRR350017.sra |
|  |  | 3-4d pupa | SRR350018 | ftp://ftp-trace.ncbi.nlm.nih.gov/sra/sra-instant/reads/ByRun/sra/SRR/SRR350/SRR350018/SRR350018.sra |
|  |  | Adult males | SRR350021 | ftp://ftp-trace.ncbi.nlm.nih.gov/sra/sra-instant/reads/ByRun/sra/SRR/SRR350/SRR350021/SRR350021.sra |
|  |  | Adult females | SRR350019 | ftp://ftp-trace.ncbi.nlm.nih.gov/sra/sra-instant/reads/ByRun/sra/SRR/SRR350/SRR350021/ SRR350019.sra |
|  |  | Postfeeding third instars | SRR350016 | ftp://ftp-trace.ncbi.nlm.nih.gov/sra/sra-instant/reads/ByRun/sra/SRR/SRR350/SRR350021/ SRR350016.sra |
|  |  | Feeding third instars | SRR350015 | ftp://ftp-trace.ncbi.nlm.nih.gov/sra/sra-instant/reads/ByRun/sra/SRR/SRR350/SRR350021/ SRR350015.sra |
|  |  | Embryos | SRR350014 | ftp://ftp-trace.ncbi.nlm.nih.gov/sra/sra-instant/reads/ByRun/sra/SRR/SRR350/SRR350021/ SRR350014.sra |
|  | *Rhagoletis pomonella* | Heads | SRX001531  SRX001885 | http://www.ncbi.nlm.nih.gov/sra/SRX001531  http://www.ncbi.nlm.nih.gov/sra/SRX001885 |
|  |  | Bodies | SRX001530 | http://www.ncbi.nlm.nih.gov/sra/SRX001530 |
|  |  | Pupa | SRX001529 | http://www.ncbi.nlm.nih.gov/sra/ SRX001529 |
|  |  | Larvae | SRX001121 | http://www.ncbi.nlm.nih.gov/sra/ SRX001121 |
|  | *Aedes albopictus* | Oocytes under non-diapause-inducing (NDI) conditions | SRR329457 | ftp://ftp-trace.ncbi.nlm.nih.gov/sra/sra-instant/reads/ByRun/sra/SRR/SRR329/SRR329457/SRR329457.sra |
|  |  | Oocytes under diapause-inducing (DI) conditions | SRR329456 | ftp://ftp-trace.ncbi.nlm.nih.gov/sra/sra-instant/reads/ByRun/sra/SRR/SRR329/SRR329456/SRR329456.sra |
|  | *Culex quinquefasciatus* | Larvae | SRR364515 | ftp://ftp-trace.ncbi.nlm.nih.gov/sra/sra-instant/reads/ByRun/sra/SRR/SRR364/SRR364515/SRR364515.sra |

**Table S2 insects’ transcriptome raw data (continued)**

| Order | Species | Sample | SRA accession number | URL |
| --- | --- | --- | --- | --- |
| Coleoptera | *Dendroctonus ponderosae* | Antenna | SRX132062 | http://www.ncbi.nlm.nih.gov/sra/SRX132062 |
|  |  | Juvenile hormone treatment | SRX132064 | http://www.ncbi.nlm.nih.gov/sra/ SRX132064 |
| Hemiptera | *Nilaparvata lugens* | Egg, larvae, pupa and adult | SRR064533 | ftp://ftp-trace.ncbi.nlm.nih.gov/sra/sra-instant/reads/ByRun/sra/SRR/SRR064/SRR064533/SRR064533.sra |
|  | *Oncopeltus fasciatus* | Embryos | SRR057571 | ftp://ftp-trace.ncbi.nlm.nih.gov/sra/sra-instant/reads/ByRun/sra/SRR/SRR064/SRR064533/SRR064533.sra |
|  |  | Ovary | SRR057570 | ftp://ftp-trace.ncbi.nlm.nih.gov/sra/sra-instant/reads/ByRun/sra/SRR/SRR057/SRR057570/SRR057570.sra |
|  | *Bemisia tabaci* | MEAM | SRR059302 | ftp://ftp-trace.ncbi.nlm.nih.gov/sra/sra-instant/reads/ByRun/sra/SRR/SRR059/SRR059302/SRR059302.sra |
|  |  | ZHJ1 | SRR062575 | ftp://ftp-trace.ncbi.nlm.nih.gov/sra/sra-instant/reads/ByRun/sra/SRR/SRR062/SRR062575/SRR062575.sra |
|  |  | MED | SRR039231 | ftp://ftp-trace.ncbi.nlm.nih.gov/sra/sra-instant/reads/ByRun/sra/SRR/SRR039/SRR039231/SRR039231.sra |
|  |  | Salivary gland | SRR316271 | ftp://ftp-trace.ncbi.nlm.nih.gov/sra/sra-instant/reads/ByRun/sra/SRR/SRR316/SRR316271/SRR316271.sra |
