# Supplementary material for: iPathCons and iPathDB: an improved insect pathway construction tool and the database
Source: Database (Oxford). 2014 Nov 10;2014:bau105. doi: 10.1093/database/bau105 (PMC4227299; doi:10.1093/database/bau105)
Supplement: Supplementary Data [file supp_bau105_Supplementary_Table_3.doc]

**Table S3 the** **assemble results of transcriptome**

| Order | Species | Contigs | Mean length of contigs (bp) | N50 |
| --- | --- | --- | --- | --- |
| Hymenoptera | *Apis cerana cerana* | 50373 | 760 | 1238 |
| Diptera | *Lucilia sericata* | 146250 | 525 | 661 |
|  | *Rhagoletis pomonella* | 13071 | 382 | 444 |
|  | *Aedes albopictus* | 48422 | 569 | 680 |
|  | *Culex quinquefasciatus* | 20308 | 487 | 580 |
| Lepidoptera | *Galleria mellonella* | 40035 | 302 | 296 |
|  | *Chilo suppressalis* | 37040 | 496 | 576 |
|  | *Spodoptera exigua* | 153619 | 605 | 580 |
|  | *Manduca sexta* | 67902 | 498 | 578 |
|  | *Melitaea cinxia* | 195086 | 138 | 128 |
|  | *Plutella xylostella* | 456026 | 685 | 954 |
|  | *Zygaena filipendulae* | 42735 | 278 | 289 |
| Coleoptera | *Dendroctonus ponderosae* | 25360 | 812 | 1135 |
| Hemiptera | *Nilaparvata lugens* | 36748 | 477 | 558 |
|  | *Oncopeltus fasciatus* | 4863 | 262 | 302 |
|  | *Bemisia tabaci* | 54860 | 541 | 669 |
